# The scientific game: Strategic Behavior and Incentives in Science

**DOI:** 10.1038/s44319-026-00900-7

**Published:** 2026-08-10

**Authors:** Lutz Bornmann, António Osório

**Affiliations:** 1 Max-Planck-Society, Administrative Headquarters, Munich, Germany; 2 Department of Economics, Universitat Rovira i Virgili and ECO-SOS, Av. Universitat1, Reus, Spain

**Keywords:** History & Philosophy of Science, Science Policy & Publishing

## Abstract

Using game theory, we demonstrate how performance metrics in academic science function as a non-monetary currency that solves the principal-agent problem in science and extracts high efforts from researchers. In light of increasingly scarce resources, these incentives require structural redesign of their mechanism to prevent strategic manipulation.

**Figure:**
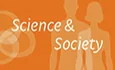

Game theory is a mathematical framework to analyze strategic interactions among rational decision-makers. It models scenarios—“games”—in which an individual's success, that is, the “payoff” or utility, depends not only on their own choices but also on the choices made by other participants. In the sociology of science and science of science studies, game theory provides a crucial analytical tool. Rather than merely describing systemic problems, it helps us to understand why researchers act the way they do by revealing how formal incentives—such as funding mechanisms, career progression, and publication metrics—shape their behavior in collaborations, publishing, and funding applications.

Several early publications lie at the intersection of the sociology of science, economics, and game theory. For example, Stephan (1996) treats scientific activity as a strategic environment structured by incentives, finance, and competition. Dasgupta and David (1994) conceptualize science as a reputation-based reward system organized around priority, disclosure, and repeated strategic interaction. These factors mentioned by the three authors are relevant for understanding the contemporary scientific ecosystem, which is characterized by unprecedented scarcity of financial resources, continuous evaluation cycles, and hyper-competitive pressures.

This article offers a conceptual analysis of several recurring features of scientific practice. It uses game-theoretic concepts to clarify how institutional incentive structures shape strategic behavior across different domains of scientific practice and to better understand contemporary structural challenges—from researchers’ avoidance of competition to strategic metric maximization to the vulnerabilities of the peer review system. By viewing science through a game-theoretic lens, it becomes clear that many behavioral distortions in academia are not isolated ethical failures, but rational strategic adaptations. Altering scientists’ behavior would therefore require more than just normative appeals; it demands a redesign of the rules of the game itself.

“By viewing science through a game-theoretic lens, it becomes clear that many behavioral distortions in academia are not isolated ethical failures, but rational strategic adaptations.”

## Winner-takes-all: sunk costs and discouragement effects

In some institutional arenas, science functions as a zero-sum or near-zero-sum game in which one researcher’s success equals another one’s loss. The zero-sum nature of the scientific game becomes most evident when scientists compete for funding opportunities. While some researchers receive funding, many others come away (nearly) empty-handed. This context also functions as all-pay auctions: every researcher pays the ‘bid’—the time, resources, and effort spent conceptualizing a proposal—but only a minority receives the payoff (Schweiger et al, 2024). The losers forfeit their investment. However, all else being equal, this structure tends to optimize for maximum effort and a high volume of submissions, because money is needed for research, and raising funds boosts reputation and improves career prospects.

“The zero-sum nature of the scientific game becomes most evident when scientists compete for funding opportunities.”

Publishing in top journals is another factor crucial for career progression, which is why many strive to do so. Publication in journals with prestigious brand names and high journal impact factors (JIFs, used as a proxy for quality), brings recognition, citations and career rewards, motivating many researchers to compete. Here, too, one of the research system’s greatest disadvantages is the cumulative waste of resources among the losing researchers, given the low acceptance rates of prestigious journals. It is fitting that Stephan (1996) characterizes the system as a ‘winner-takes-all’ tournament. He illustrates this using the pursuit of ‘being the first’ as an example: priority goes only to the first discovery, while the runner-up receives little or no payoff, even if the underlying work is of similar or even better quality.

However, the assumption that zero-sum games and all-pay auctions always maximize overall effort must be nuanced, particularly when the playing field is skewed. In game theory, this dynamic is known as the discouragement effect within asymmetric contests. Researchers do not enter grant competitions or submission processes for high-impact journals with equal chances. When the environment is dominated by well-known researchers or elite institutions, the strategic incentives shift. If less established researchers feel that their chances of success against these prominent players are very low, their rational choice can be to skip the contest entirely. This behavior is driven by the sunk-cost nature of proposal writing or the extensive efforts required for getting accepted by top-tier journals.

Thus, asymmetric zero-sum environments do not inevitably incentivize community-wide effort and diversity. Instead, they may trigger a discouragement effect that disincentivizes participation from a broader, diverse pool of scientists. Over time, this mechanism exacerbates the Matthew effect: the contest narrows to a small and well-resourced group of players, stratifying the scientific community and favoring already advantaged candidates (Stephan, 1996).

## The consequences of high-powered incentives in science

Many researchers face uncertain employment conditions, and the path to a permanent position requires accumulating core resources such as research grants and publication in top journals that are scarce and difficult to obtain. From a game-theory perspective, competition for these resources operates as a mechanism that increases scientists’ efforts with significant benefits, spillovers, and externalities for the knowledge-building process and for society despite the limited resources that society makes available for research. As a result of these efforts, the global scientific output has experienced high growth rates over the years (Bornmann et al, 2021).

However, when competitive pressure exceeds a critical threshold and evolves into a hyper-competitive environment, the behavior can change. Scientists no longer focus on the research itself—their foundational or existential motive—but rather on the criteria used to measure their performance, such as evaluation metrics. In other words, research activity is transformed into a strategic game of metric maximization, which manifests in inflated publication strategies, manipulative self-citations, or research misconduct. For instance, Fong and Wilhite (2017) demonstrate that publication pressure induces strategic manipulation, such as padded citations (authors superfluously add citations to reference lists to boost their own metrics) and honorary authorships (listing someone who did not make a substantial contribution), to inflate performance metrics. Another form of strategic manipulation occurs when authors cite reviews published in prestigious journals instead of the primary literature, making the reference list appear more impressive, avoiding an in-depth literature review, and diverting recognition away from the original sources (Tahamtan and Bornmann, 2019).

“… research activity is transformed into a strategic game of metric maximization, which manifests in inflated publication strategies, manipulative self-citations or research misconduct.”

Smaldino and McElreath (2016) show that a research system rewarding publication rate and prestige incentivizes behaviors that maximize publishable output while increasing the danger of reducing methodological rigor, even without individual intent. These incentives operate through two different but often reinforcing metrics: the number of papers published and the JIF of the journals in which they are published. Thus, researchers are encouraged not only to publish more but also to publish in journals with a high JIF. Higginson and Munafò (2016) present a mathematical optimization model showing that, under current incentive structures, the strategy that maximizes a researcher’s career fitness is to allocate resources across multiple small, underpowered studies rather than to fewer, more rigorous and substantive ones. This strategic allocation of effort maximizes publication volume but inherently reduces statistical power, thereby systematically inflating the rate of erroneous, weak, and non-replicable publications.

### An optimal balance

The counter-model to a hyper-competitive system can be described as the Insulated Science Model. This environment is defined by highly secure employment conditions, such as institutional core funding and tenured positions, which reduce existential competitive pressure. The incentives for strategic gaming or deception decrease because long-term income is guaranteed independently of short-term output. Science potentially benefits from reliable and robust findings, as researchers can pursue long-term projects without immediate publication and funding pressure. Yet, absolute job security and the lack of pressure may create a moral hazard problem, where insulation from professional risk diminishes a researcher’s incentive to maintain high levels of effort. When stability is absolute, extrinsic incentives can weaken, potentially reducing overall research activity and diminishing the competitive drive to publish in reputable venues and to search for funding.

An optimal scientific architecture probably requires a design that establishes a middle ground between the hyper-competitive environment and the Insulated Science Model. As Manso (2011) demonstrates in an economic analysis of innovation, motivating novel discoveries requires an incentive scheme that tolerates short-term failure by providing job and financing security, while rewarding long-term success through competitive payoffs. Initiatives to reform research assessments, such as the Leiden Manifesto (Hicks et al, 2015) and the Metric Tide (Wilsdon et al, 2015), attempt to shift the payoff matrix and incentives toward the responsible use of metrics in research evaluation, in order to sustain a productive and competitive environment without incentives for gaming.

### The rationality of cooperation

In game theory, the state of competitive frameworks where no individual can improve their payoff without worsening another’s is defined as the Pareto Optimum. It represents the highest possible benefit for all. Although Pareto outcomes are often desirable, they are not necessarily unique, nor do they always maximize equality for everybody. The other extreme of competitive frameworks may trap individuals in a classic Prisoner’s Dilemma. This describes a strategic scenario in which individual actors face a strong incentive to act uncooperatively (to defect), even though cooperation would yield a better collective outcome for all participants. The Prisoner’s Dilemma can culminate in a suboptimal Nash Equilibrium: a state in which no individual has an incentive to unilaterally change their strategy because any deviation would worsen their immediate position. In an isolated scientific contest, this could mean, for instance, that researchers, fearing a loss of priority (scooping), hoard data and resources rather than sharing them, or engage in free riding on each other’s efforts, thereby missing the collective Pareto optimum.

The need to overcome the suboptimal equilibrium is driven by structural dependencies in the science system. As the complexity and financial costs of modern empirical research rise, and as many societal problems can only be solved through interdisciplinary dialogue, researchers can no longer maximize their payoffs in isolation. The necessity to cooperate is intensified by institutional incentives to maximize publication volume. Thus, the ‘publish or perish’ pressure paradoxically strengthens the structural dependencies between actors, forcing them to cooperate. The cooperation shift is evident in the division of scientific labor. Powell et al (2005) argue that modern science evolves through dense networks, which serve as primary sources of opportunity and influence. Larivière et al (2021) utilized the Contributor Roles Taxonomy (CRediT) to demonstrate how scientific collaboration has evolved into highly specialized roles. By dividing tasks—such as conceptualization, data curation, and formal analysis—across diverse teams, researchers optimize their collective payoff matrix and their ability to tackle scientific problems that exceed individual capacities.

“… the ‘publish or perish’ pressure paradoxically strengthens the structural dependencies between actors, forcing them to cooperate.”

But how does the system prevent individual researchers from taking advantage of their colleagues in collaborative projects? For example, researchers might take credit for the work of others while contributing very little to the project themselves. The mechanism that transforms scientific competition into scientific cooperation is the transition from a single interaction to an iterated Prisoner’s Dilemma. It occurs when the same actors interact repeatedly over time, introducing the ‘shadow of the future’—where the expectation of future encounters and evaluations shapes strategic choices. In a single, one-shot game, uncooperative behavior carries no long-term consequences for the defecting actor. In an iterated game, however, current defection triggers future retaliation, while cooperation fosters reciprocal altruism because actors know they will depend on each other in subsequent rounds. For instance, in the case of the first publication from a project involving several researchers, it may be possible that a researcher contributes little to the publication. However, to continue collaborating with the project team, the researcher should clearly signal that they will be heavily involved in the second and subsequent publications.

Because past behavior is not only visible within a project team but also to other actors within scientific fields, a researcher’s reputation operates as a critical economic asset. A researcher who defects in publications by withholding data or information from a project, for example, damages this asset and signals unreliability. Word of such behavior gets around. Other network participants respond in the iterated game by excluding the uncooperative actor from future collaborations, directly diminishing long-term publication and funding success. Therefore, information sharing and resource pooling, as modeled by Haeussler et al (2014), do not stem from moral altruism. Instead, they function as strategic, costly signals of reliability that protect a researcher’s status and ensure that long-term individual payoffs are maximized through immediate cooperation (Dasgupta and David, 1994).

### The credit economy: co-authorship dynamics and micro-mechanism design

In game theory, the viability of any cooperative agreement depends on the alignment of players’ payoffs. Within the academic ecosystem, this payoff is denominated in citation credit, authorship prestige, or the possibility of future cooperation. A structural friction emerges because the academic reward system disproportionately concentrates credit on the first and last authors, leaving the expected payoff for middle authors close to zero. Rational strategic actors will therefore not allocate high effort if their expected payoff is negligible. This credit asymmetry is empirically documented by Shen and Barabási (2014), who demonstrate that collective credit allocation in multi-author papers is highly skewed, concentrating recognition within a limited subset of contributors. The uneven distribution becomes problematic as modern research increasingly relies on large, multi-institutional collaborations. While contributorship data, such as the CRediT taxonomy, make the underlying division of labor visible, conventional authorship order frequently obscures these individual contributions.

To resolve distributional conflicts and stabilize collaborative teams, researchers utilize localized game-theory solutions. One strategy involves entering into repeated cooperative games of indirect reciprocity, where co-authors explicitly agree to alternate first and last authorship positions across a series of publications over time to balance the long-term payoff matrix. However, when authorship positions are rigid or non-exchangeable, the stability of the team requires side payments—compensations outside the immediate game that restore the incentives for middle authors. Within research groups, principal investigators (PIs) can act as managers of these side payments, offering alternative payoffs such as future employment, funding, targeted letters of recommendation, or exclusive access to specialized laboratory infrastructure.

The management and distribution of payoffs position the PI as an institutional mechanism designer. Mechanism design involves reverse-engineering a strategic environment by defining a specific objective and adjusting the rules of the game to ensure that players’ rational choices lead to that objective. For example, a PI aiming to maximize the output of a multi-stage project should pre-define an incentive structure of alternating authorships and side payments to sustain collective motivation and collaboration. As synthesized by Zollman (2018) and derived from the credit allocation models of Shen and Barabási (2014), if the internal reward architecture is poorly aligned, cooperation becomes fragile, leading to strategic distortions or the collapse of team productivity.

### Public goods and the volunteer’s dilemma

The social and community-oriented ideal underlying the scientific enterprise model resembles a Public Goods Game: a scenario where a resource, such as quality control or high-quality publications, is accessible to all—in this case, the authors of manuscripts and the readers of publications—regardless of their individual contribution. Many critical processes in academia—such as publishing, peer review, editorial positions, mentoring, and committee work—rely heavily on uncompensated labor. In contrast to the private sector, where services are strictly commodified, the reliance on free labor has become institutionalized within the scientific system, which is partly justified by the fact that modern research is predominantly funded by public money and that society expects a return on investment in terms of innovation and public services. Monetizing essential academic duties carries the profound risk of violating the scientific norm of disinterestedness, however. In the sociology of science, disinterestedness—one of the four core institutional imperatives formulated by Merton (1973)—dictates that scientists conduct research for the benefit of a common enterprise, rather than for personal, financial, or ideological gain. It mandates institutional control of motives, ensuring that the pursuit of knowledge remains insulated from individual self-interest. Thus, providing academic services for free is not merely an economic quirk, but a normative duty designed to preserve the integrity of the system.

“… providing academic services for free is not merely an economic quirk, but a normative duty designed to preserve the integrity of the system.”

However, like any Public Goods Game, it is inherently vulnerable to free riding. The Open Access movement illustrates this dynamic: while it has drastically expanded science as a public good in recent years, it simultaneously allows external economic actors to profit from a continuous flow of publications, without ever contributing back to the public pool. A related outcome is the rise of predatory publishers within the Open Access ecosystem (Grudniewicz et al, 2019). If the publication is financed through article processing charges, publication volume becomes directly linked to editorial revenues and profits. This creates incentives for some publishers to maximize the number of accepted papers while saving on editorial scrutiny and peer review. In this way, Open Access can generate a perverse economy in which the argument of accessibility and public knowledge is used to extract rents from researchers, institutions, and public funding systems without contributing meaningfully to scientific quality or the cumulative stock of knowledge.

In game-theoretic terms, these actors around the Open Access ecosystem are classic free riders, extracting the payoff of the scientific community’s labor without paying any or only minimal initial costs. To prevent systemic exploitation, the structural organization of knowledge becomes critical. As Murray and O’Mahony (2007) demonstrate, the central issue for sustaining cumulative innovation is not a binary choice between ‘open’ or ‘closed’ knowledge. Instead, the challenge lies in designing institutional mechanisms that organize access, reuse, and reciprocity in a way that ensures that all participants are actively incentivized to build upon and replenish the public pool of prior work with high-quality work.

One of the most important services that scientists provide for free is peer review. The process operates as a classic Volunteer’s Dilemma—a specific iteration of the Public Goods Game. In this scenario, a critical public good—scientific quality control—is only sustained if enough individuals willingly absorb the private cost. In academia, this cost is the time and cognitive effort diverted from one’s own research to evaluate the work of others. Since the system is formally unrewarded, the rational, short-term strategy is to free ride: to submit their own papers while declining review requests, assuming others will shoulder the burden. If all actors adopt this strategy, the system collapses. The model only survives because it is underpinned by generalized reciprocity and alternative, non-monetary payoffs. For instance, editorial positions circumvent the Volunteer’s Dilemma by compensating actors with structural influence, network visibility, and recognition. This aligns individual strategic interests with the provision of the public good.

However, relying on unrewarded, volunteer-based architecture introduces structural vulnerabilities, particularly regarding the integrity of the evaluation process. Because a reviewer’s actions are largely unobservable and shielded by anonymity, the process generates a structural moral hazard. When a researcher is tasked with reviewing a direct competitor, this insulation from accountability provides a strategic opportunity to act opportunistically—for example, by delaying the review or unfairly criticizing the manuscript to protect the reviewer’s own stakes in the zero-sum game. Conversely, evaluating a known colleague can incentivize reciprocal cronyism. In these instances, the reliance on uncompensated volunteers, combined with a lack of formal oversight, risks transforming peer review from an objective mechanism of quality control into an arena compromised by strategic self-interest. In response to this problem, an increasing number of publishers, such as *EMBO* *Press* or *eLife* (Mutz et al, 2025), have introduced transparent review. These practices include making reviewer reports, editorial decisions (comments), and author responses publicly available, thereby reducing the unobservability of evaluation and increasing accountability.

### Discussion

When viewed through a game-theoretic lens, the contemporary scientific enterprise reveals a tension between individual rationality and collective efficiency. The structural challenges currently facing the scientific community—ranging from strategic metric maximization to misconduct in peer review—are not anomalous ethical deviations. Rather, as demonstrated by the formal models of Smaldino and McElreath (2016) and Higginson and Munafò (2016), these behaviors represent rational strategic adaptations to poorly aligned incentives and payoff matrices within a hyper-competitive, resource-scarce ecosystem.

However, game theory also provides the theoretical blueprint to resolve these dilemmas. The natural emergence of cooperation within iterated scientific networks demonstrates that structural interdependencies and the ‘shadow of the future’ can suppress opportunistic behaviors. In this context, reputation functions as an economic asset that penalizes uncooperative behavior at the macro and aggregate level, while PIs can stabilize collaborations at the micro and individual level through localized mechanism design and strategic side payments. These counter-mechanisms prove that scientists are responsive to structural incentives.

Achieving a superior Pareto Optimum across the scientific community requires a transition from passive observation to active incentives and systemic mechanism design. Science cannot be reformed by moral directives or normative appeals alone. To foster an ecosystem that prioritizes verifiability, a robust division of labor, and knowledge sharing, the structural rules of the game would have to be rewritten.

The conventional critique of performance metrics often oversimplifies their systemic role by framing them purely as destructive forces. From a game-theoretic and mechanism-design perspective, quantitative metrics and journal prestige serve a highly functional purpose: they act as a robust, non-monetary currency that effectively solves the principal-agent problem inherent in academic research. Because direct financial compensation for individual discoveries is rare, and the Insulated Science Model risks moral hazard, the system requires a powerful mechanism to incentivize high levels of effort. The intense drive to publish in prestigious, highly cited venues forces researchers to exert massive cognitive labor, conduct complex experiments, and dedicate extensive resources, even in the absence of direct monetary rewards. In what other area of society besides science—such as medical practice or the legal system—are there goals that are highly regarded but not linked to direct financial incentives? In this sense, the metric-driven evaluation system is astonishingly successful at its primary task: it maximizes aggregate scientific output and extracts extraordinary effort from rational actors.

The practical challenge is not to abolish established metrics, but to recalibrate the rules of the game to ensure they reward genuine rigor rather than strategic manipulation. In game-theoretic terms, the system should reduce the asymmetry of information between the researcher and the evaluator. Historically, scientific claims were verified through live demonstrations in front of peers—a highly observable form of early quality control that made deception practically impossible. In modern science, this principle of absolute observability could be digitally reinstated. To access the high payoffs associated with elite journal publications and subsequent citation accumulation, researchers could be required to submit their entire analytical pipeline, including raw data, code, and methodological protocols. Mechanism design can then leverage emerging technologies, such as artificial intelligence and automated computational auditing, to rigorously recalculate and verify the submitted findings prior to publication. By conditioning the traditional rewards of impact and prestige on radical transparency and algorithmic verification, the strategic cost of ‘gaming’ the system becomes prohibitively high. This practical recalibration ensures that the rational pursuit of high-profile metrics strictly aligns with the production of robust, reproducible science.

“The practical challenge is not to abolish established metrics, but to recalibrate the rules of the game to ensure they reward genuine rigor rather than strategic manipulation.”

## Supplementary information

Peer Review File
