# Supplementary material for: The scientific game: Strategic Behavior and Incentives in Science
Source: EMBO Rep. 2026 Aug 10;27(18):5172–7. doi: 10.1038/s44319-026-00900-7 (PMC13601502; doi:10.1038/s44319-026-00900-7)
Supplement: Supplementary file 1 [file 44319_2026_900_MOESM1_ESM.pdf]

EMBO reports

The Scientific Game

Lutz Bornmann and António Osório

Corresponding author(s): António Osório (antonio.osoriodacosta@urv.cat)

Review Timeline:

Submission Date:	3rd Aug 26
Accepted:	5th Aug 26

Editor: Holger Breithaupt

Transaction Report: This article was editorially reviewed.
